# Evolutionarily recent retrotransposons contribute to schizophrenia

**DOI:** 10.21203/rs.3.rs-2474682/v1

**Published:** 2023-01-23

**Authors:** Giorgia Modenini, Paolo Abondio, Guia Guffanti, Alessio Boattini, Fabio Macciardi

**Affiliations:** University of Bologna

**Keywords:** schizophrenia, retrotransposons, eQTL, evolution, polymorphic TEs

## Abstract

Transposable Elements (TEs) are mobile genetic elements that constitute half of the human genome. Recent studies suggest that polymorphic non-reference TEs (nrTEs) may contribute to cognitive diseases, such as schizophrenia, through a cis-regulatory effect. The aim of this work is to identify sets of nrTEs putatively linked to an increased risk of developing schizophrenia. To do so, we inspected the nrTE content of genomes from the Dorsolateral Prefrontal Cortex of schizophrenic and control individuals, and identified 38 nrTEs which possibly contribute to the emergence of this psychiatric disorder. Furthermore, we performed in silico functional inferences and found, for instance, that 9 of the 38 nrTEs act as expression/alternative splicing quantitative trait loci (eQTLs/sQTLs) in the brain, suggesting a possible role in shaping the human cognitive genome structure. Therefore, to our knowledge, this is the first attempt at identifying polymorphic nrTEs that can contribute to the functionality of the brain. Finally, we suggest that a neurodevelopmental genetic mechanism, which involves evolutionarily young nrTEs, can be the key to understanding the ethiopathogenesis of this complex disorder.

## Introduction

Transposable Elements are DNA sequences which have the ability to move around in the genome. TEs constitute 53–60% of the human DNA^1,2^ and are essential elements in driving genome evolution^3^. Among non-LTR retrotransposons (Alu, LINE and SVA), only LINE-1 (L1) can actively transpose, while Alu and SVA rely on L1’s machinery to mobilize themselves^4^. While the vast majority of TEs are no longer transpositionally active, they can still play a functional role as exapted enhancers or transcriptional start sites^5–8^, by inserting Transcription Factor Binding Sites (TFBS)^9,10^ or by acting as novel RNA genes such as long non-coding RNAs (lnc-RNAs)^11^. Therefore, TEs participate in regulating the expression of nearby genes, at transcriptional and post-transcriptional levels, providing a crucial role as both cis- and trans-regulatory RNA sequences^12^. Baillie and colleagues^13^ also found that protein-coding loci are disproportionately affected by TEs, with over-representation of L1s in introns and Alus in exons. Overall, TEs seem to predominantly affect neurogenesis and synaptic function, with studies suggesting a putative regulatory role of TEs in the neural genome^14–18^. Nonetheless, only initial research work has been systematically performed on this issue and the TE-controlled regulatory architecture of the human genome still needs to be better explored and investigated. According to recent studies, TEs’ insertion polymoprhisms “can be mapped as cis- expression quantitative trait loci with substantial effects on gene expression, especially at loci involved in immune response and cognitive function”^19,20^. A polymorphic TE insertion can be exapted as a functional element depending on its site of insertion within the genome, or it can disrupt an already existing enhancer^21^. Additionally, polymorphic TEs have been shown to be closely associated with complex phenotypes in GWAS investigations suggesting that polymorphic non-reference TEs (nrTEs) may contribute to disease phenotypes through cis- regulatory effects^22–24^. Interestingly, nrTEs are relatively young compared to fixed TEs, therefore they are likely to have had a role in the most recent phases of the evolution of our species, which particularly involved the brain and superior cognitive abilities.

In recent years, mounting data provided evidence that epigenetic mechanisms and TEs are playing a key role in schizophrenia and other neurological disorders^17, 25–27^. For example, Bundo and colleagues^15^ described an increased number of somatic L1 retrotransposition in the Dorso-Lateral PreFrontal Cortex (DLPFC, Brodmann’s area 46) of people affected by schizophrenia, observing that the total number of brain-specific L1 insertions tended to be higher in schizophrenia patients, an observation confirmed by Doyle et al.^18^. In our own previous work^17^ and in a recent review^28^, L1 insertion sites were also reported to be preferentially localized to synapse- and schizophrenia-related genes. Guffanti et al.^25^ developed a method to quantify the tissue-specific expression of TEs (as well as other ncRNAs), and found more than 650,000 expressed TEs in the DLPFC of post-mortem human brains: about 114,000 expressed TEs are differentially expressed between schizophrenia cases and healthy controls and mostly represented by primate- or human-specific elements.

A recent study suggests another potential key role for TEs in rewiring the local functional architecture of Human Accelerated Regions (HARs) in Schizophrenia and Bipolar Disorder^29^. Indeed, HARs have been implicated in neurodevelopmental and neuropsychiatric disorders^30–32^, and most HARs are known to act as developmental enhancers that are involved in controlling and regulating human cognition^33–37^.

In this study, our goal was to identify polymorphic TEs that can potentially contribute to schizophrenia. Inspecting the non-reference TE content of DLFPC genomes of schizophrenic individuals (SCZ), we will 1) compare SCZ with control (CTRL) genomes; 2) check the population-specific/geographic distribution of the identified variants; 3) perform haplotype-based association tests; 4) in silico explore the nrTEs possible functional roles as cis-regulatory elements of protein-coding genes and as putative modifiers of known HARs.

## Subjects And Methods

DNA from the DLPFC of ten schizophrenic patients and ten psychiatrically healthy controls has been obtained from the UCI Brain Bank, following an UCI/IRB approved protocol.

DNA from the 20 DLPFC samples has been aligned to the human reference genome hs37d5 (http://ftp.1000genomes.ebi.ac.uk/vol1/ftp/technical/reference/) with BWA-mem^38^. After sorting and merging with Samtools^39^, the GATK best practices were applied to generate VCF files that include SNPs as well as Indels (https://gatk.broadinstitute.org/hc/en-us/articles/360035894711-About-the-GATK-Best-Practices).

We searched for non-reference TEs (nrTEs: Alu, LINE1 and SVA) with the Mobile Element Locator Tool (MELT) v.2.1.5^40^, using MELT-Split with default parameters on our twenty high-coverage genomes (Supplementary Table 1). To analyze the geographic variability of the putative schizophrenia-related nrTEs, we additionally selected 125 samples from the “1000 Genomes Project” phase 3^41^. These 125 samples were analyzed with MELT jointly with SCZ and CTRL samples. Only “PASS” sites were included in a single final VCF file and only nrTEs mapping in genic or regulatory regions (introns, exons, promoters, terminators and UnTranslated Regions, UTRs) on autosomal chromosomes were considered for further analyses. Fisher tests of independence were performed to identify which nrTEs show significantly different frequencies in SCZ and CTRL. Tests were performed with one and two degrees of freedom, respectively, for allelic and genotype frequencies. nrTEs that yielded nominally significant tests (pval < 0.05) at least for allele and/or genotype frequencies were considered as putatively related with schizophrenia.

To assess the genetic relationships among the individuals included in our dataset, as well as their ancestry, we implemented a principal component analysis (PCA) and ADMIXTURE analysis^42^, both on the whole variant dataset (single nucleotide polymorphisms, SNPs, and nrTEs) and the nrTE-based only. Quality control (QC) was performed with the PLINK software^43^.

We also performed a haplotype reconstruction procedure with SHAPEIT v.1.9^44^ on the whole variant dataset to contextualize the genotyped nrTEs into their local genetic environment and evaluate the frequency of the corresponding haplotypes within the DLPFC cohort. We then performed an association test using Beagle v.3.3.2^45^ on the nrTEs with significantly different allelic and/or genotype frequencies between SCZ and CTRL.

As highlighted in the introduction, TEs can act as *cis* regulatory elements by, for example, modifying the expression of nearby genes and inducing alternative splicing. Therefore, we checked if non-reference TEs may act as eQTLs and/or sQTLs, by comparing our significant results with those from Cao et al.^46^, based on the GTEx dataset^47^.

Moreover, we verified whether the statistically significant non-reference TEs (i.e., nrTEs with significantly different allele/genotype frequencies between cases and controls) are located close to genes previously studied in the context of schizophrenia.

We also compared our 38 nrTEs with the lists of HARs as originally identified by Pollard et al.^48,49^, Prabhakar et al.^50^, Bird et al.^51^, Capra et al.^52^ and Gittelman et al.^53^, to check if some of the identified TEs are located in those regions.

## Results

### non-reference retrotransposon insertions

We identified 7 952 nrTEs in genic/regulatory regions: 6 542 Alu (82.3%), 1 065 LINE-1 (13.4%) and 345 SVA (4.3%), as shown in Table 1, using MELT-Split on our 145 samples (10 SCZ, 10 CTRL and 125 normal individuals from 1000 Genomes Project, 1KGP).

We checked the chromosomal distribution of the 7 952 nrTEs and found no significant difference (Fisher pval > 0.63) between the expected and observed content of the different families of TEs (SVA, LINE-1 and Alu) (Supplementary Figs. 1–3).

### Population Structure Of The Dataset

**Figure 1:**
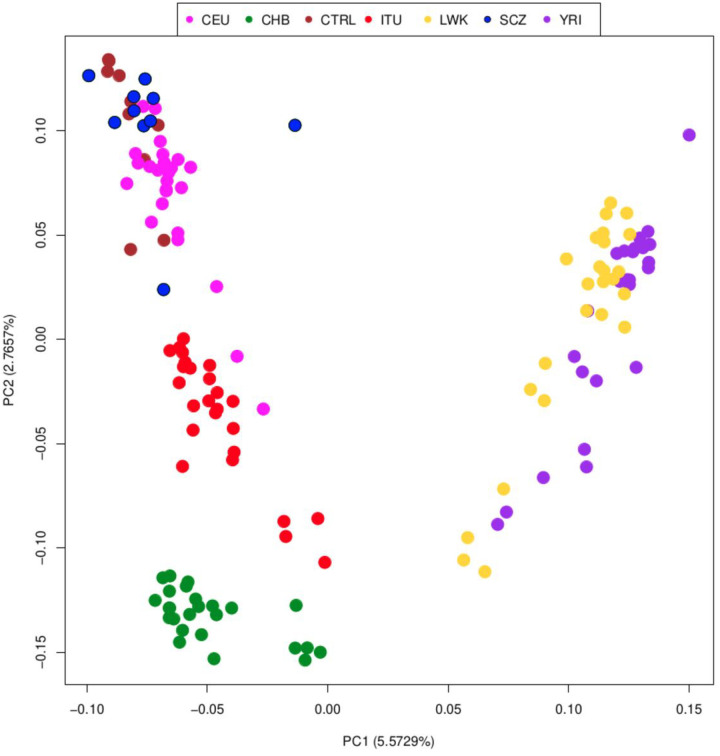
Principal Component Analysis (PCA) of the DLPFC and 1KGP samples based only on non-reference TEs. Pink: Europeans (CEU); green: Han Chinese in Beijing (CHB); brown: controls from the DLPFC (CTRL); red: Indian Telugus (ITU); yellow: Luhya in Kenya (LWK); blue: schizophrenic individuals from the DLPFC (SCZ); violet: Yoruba in Nigeria (YRI).

**Figure 2:**
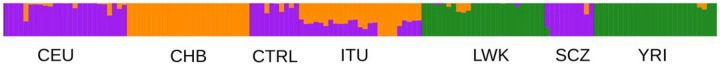
ADMIXTURE plot based only on nrTEs. K=3 is shown (CV error = 0.37350).

To contextualize our 20 DLPFC samples in the worldwide genomic landscape, we performed PCA and ADMIXTURE analyses on nrTEs genotypes and found that the best estimate for K in the latter is 3, with CV error = 0.37350, including 125 1KGP samples from 5 populations. Our results (Figs. 1 and 2) show that nrTEs are useful predictors of the genomic structure of the different human populations, as confirmed by the intermediate position of Indians (ITU) between Europeans (CEU) and Chinese (CHB), as well as by the clear differentiation between Eurasian and African samples.

The ADMIXTURE plot shows that CTRL and SCZ share the same ancestral components of CEU, (violet). Han Chinese (orange) have their own ancestral component, as well as the two African populations (colored). ITUs show a mixture of European and Asian components. These results, as a whole, are coherent with those obtained using SNPs^41,54^.

Accordingly, nrTEs show systematic differences in allele frequencies across populations: 3 131 of 7 952 non-reference TEs, 2 711 Alu (41.4%), 332 LINE-1 (31.1%) and 88 SVA (25.5%), have a significant geographic stratification (Fisher pval < 0.05) (Supplementary Table 2), with 2 263 (28%) presenting with an allele frequency > 5%. Among these, 1 501 nrTEs are found only in African populations, 833 are exclusive of non-African populations (Europeans, Indian Telugus and Chinese) and 955 are common to all the five groups (see Supplementary Table 2 and Supplementary Fig. 6). Both methods (PCA and ADMIXTURE) highlight that DLPFC samples overlap with CEU, with the partial exception of a single SCZ sample, which presents signs of admixture with a Sub-Saharan African source (as represented by YRI and LWK).

These findings suggest that SCZ and CTRL are genetically homogeneous; consequently, variants associated with the disease condition do not depend on underlying population structure.

### Comparison Between SCZ And CTRL Subjects

We then compared the distribution of allele and genotype insertion frequencies of nrTEs (herein, ‘counts’) between SCZ cases and normal CTRL. We detected 38 non-reference TEs with significantly different allele/genotype counts between cases and controls: three LINE1s, three SVAs and 32 Alus, considering only those TEs that yielded significant Fisher tests for allele and/or genotype counts^55,56^ at the nominal p-value = 0.05 (Table 2). Given the limited size of our sample, no correction for multiple testing was performed. All significant nrTEs belong to evolutionarily recent elements (L1Ta and AluY), with two exceptions: the L1 on chr12:126802943 (undetermined subfamily) and the Alu on chr7:141748320 (which belongs to the subfamily Sz, older than Y).

Of these 38 nrTEs, 11 show a significant difference in allele counts only, 14 in genotype counts only and 13 in both allele and genotype counts. Interestingly, most of these TEs also show evidence of differential segregation (Fisher test, pval < 0.05) in human populations (27 for allele counts, 24 for genotype counts, 23 for both).

**Figure 3:**
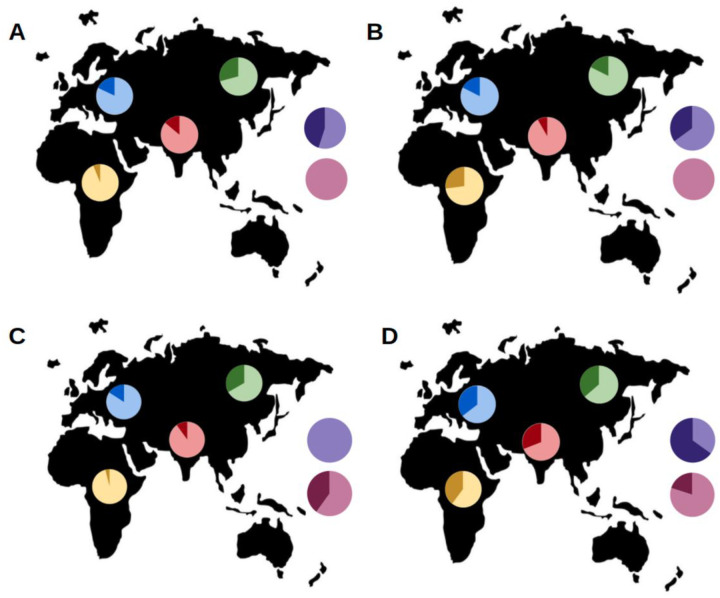
Allele frequencies of Alus on chr4:17150918 (A), chr4:23511024 (B) and chr11:40727097 (C), compared to SVA on chromosome chr20:5268423 (D). Darker colors indicate the presence of the TE (+), while lighter colors indicate the absence (−). Following populations are displayed: Europeans (blue), Indian Telugus (red), Chinese (green) and Africans (yellow), represented by Luhya in Kenya and Yoruba in Nigeria. Allele frequencies for schizophrenic individuals and healthy controls are shown in violet and pink, respectively.

The most significant allele-wise results (pval < 0.01) among the 38 significant nrTEs include three Alus and one SVA, whose insertion can be found on: chr4:17150918 and chr4:23511024 (both AluY and only observed in SCZ), chr11:40727097 (AluYa5, only observed in CTRL) and chr20:5268423 (SVA, more frequent in SCZ). The three Alus show a statistically significant geographical distribution (Fig. 3 and Supplementary Table 3), presenting variable insertion frequencies across populations, while the SVA on chr20:5268423 has similar allele frequencies in all the considered populations.

### Haplotype-based Association Analysis

We then performed haplotype-based analysis using Beagle on the previously detected 38 variants (Table 2) and obtained two significant results. The first one was for a 188bp haplotype including the AluYb on chr5:100497396, in the promoter of the gene ST8SIA4. This haplotype is characterized by 4 polymorphisms: T + TG (where “+” points out to the presence of the nrTE and the other letters represent single nucleotide variants). It is present with 15 copies in CTRL and 2 in SCZ, suggesting a strong association (pval = 6.86 × 10^−5^) between the presence of the haplotype and the absence of the disease.

The second significant result was for a 1 172 bp haplotype including the locus of the AluYb7 on chr4:23511024 in the promoter of MIR548AJ2. Interestingly, such haplotype is characterized by the absence of the insertion, with polymorphisms GC-TTI (where “I” stands for InDel and “-” indicates the absence of the nrTE); accordingly, it was found with 19 copies in CTRL and 6 copies in SCZ (pval = 3.93 × 10^−5^), while the mentioned Alu is completely absent in CTRL samples (Table 2) and present in 7 SCZ, only in an heterozygous condition.

### In-silico Functional Inferences For Non-reference TEs

Notably, at least seven genes putatively mapped by our significant non-reference TEs have been already associated with schizophrenia: LRRC4C^57^, LRRC7^58–60^, ST8SIA4^61–63^, MGAM^64^, ADAMTS1^65^, MIR548AJ2^66^ and SCN5A, which is linked to the Brugada syndrome^67–69^.

We then compared our set of 38 significant nrTEs with the eQTLs and sQTLs TEs lists produced by Cao et al.^46^ using the GTEx dataset^47^ (Supplementary Table 4). Indeed, 27 TEs (3 LINE-1 and 24 Alu) (68.4%) were detected as potential eQTLs acting in different tissues, 7 of which are expressed in the brain. As for sQTLs, 13 (34.21%) of our TEs (2 LINE-1 and 11 Alu) were detected as potentially contributing to alternative splicing in different tissues, 2 of them supposedly acting in the brain (chr11:76990585 and chr2:36476695). All sQTLs TEs were also eQTLs, with the single exception of an Alu (chr5:159122155, in the promoter of ADRA1B) which works only as sQTL.

We also compared our set of 38 nrTEs with the lists of HARs produced by other Authors (see Materials and Methods) and found that none of these nrTEs are located into HARs.

## Discussion

Far from being “junk”, it has been shown that Transposable Elements, such as non-Long Terminal Repeats retrotransposons, can contribute to human genomic diversity in various ways. Mounting data suggest both a positive and a detrimental role of retrotransposons in shaping the human cognitive traits^17^ and in the development of brain and Central Nervous System (CNS) structures^70,71^. Several authors also suggest that retrotransposons have an important role in some neurological and psychiatric disorders, such as schizophrenia^15,16,18,25,26^.

However, the full impact of TEs on the human genome is still unclear, both for technological/methodological limitations as well as for our current lack of knowledge of their precise effects and interactions with other genetic/epigenetic elements. Since a relationship between cognitive disorders and reference TEs has been the subject of several recent studies^15,25,26^ with this work, we aimed to provide a first overview of non-reference TEs that can potentially contribute to increasing the risk of developing schizophrenia.

In a first step, to evaluate whether nrTEs distribution can contribute to population substructure (= nrTEs frequency differences due to specific populations origin/evolutionary trajectory) and lead to specific population patterns, we inspected the genetic distribution of traditional DNA variants (SNPs, Indels, CNVs) and of nrTEs in our 20 DLPFC subjects together with 125 worldwide individuals that we collected from the 1KGP. Our PCA and Admixture results show that nrTEs are present with mostly population-specific frequencies within our world-wide dataset, similarly to the well-known patterns previously detected in SNP-based studies and we ultimately confirm that nrTEs too contribute to the higher genomic diversity of African populations compared to non-African’s^41,54,72^.

Our results (Figs. 1 and 2) also show that our SCZ and CTRL individuals fall within the European genomic variability (represented by CEU in both PCA and Admixture analyses) and share a predominant European ancestral component, except for a single individual showing signs of admixture with a Sub-Saharan African source.

Therefore, our results confirm both that polymorphic nrTEs can be used as reliable markers for reconstructing the genomic structure (and potentially the history) of the considered samples/populations, as previously suggested by others^40,73,74^ and that our SCZ and CTRL sample presents a clear nrTEs genetic homogeneity, which allows to exclude spurious associations due to hidden population substructure.

To our knowledge ours is the first attempt at detecting non-reference (polymorphic) TEs as possibly associated with schizophrenia. Even though our results are based on a relatively low number of subjects, spurious associations are unlikely, given the homogeneity and absence of population substructure of our SCZ/CTRL sample. Indeed, we identified 38 nrTEs whose frequencies are significantly different between Schizophrenics and Controls. Allele frequency differences for these TEs are similar or even higher than those observed between the most different ‘control’ populations for the same insertions, suggesting that it is highly improbable that the observed differences emerged by chance. Allele-wise, the most significant results (pval < 0.01) are three Alus and one SVA, which respectively fall on chr4:17150918, chr4:23511024, chr11:40727097 and chr20:5268423. The first two Alus are found only in SCZ, the third only in CTRL and the SVA on chromosome 20 is more frequent in SCZ subjects. The Alu on chr11:40727097 is completely absent in SCZ (but present in 27.4% of the other individuals) (Fig. 4C) and is located in the second intron of the Leucine Rich Repeat Containing 4C (LRRC4C) gene, which is highly expressed in the frontal cortex and has been associated with a positive response to antipsychotic therapy with lurasidone in SCZ patients^57^. In our case, the absence of this Alu insertion is preferentially associated with the schizophrenic condition (see Tables 2 and 3).

After inferring the haplotypic context surrounding the 38 nrTEs of interest, we performed an association test with Beagle, which returned two significant results: one is the AluYb on chr5:100497396, located in the promoter of the gene ST8 Alpha-N-Acetyl-Neuraminide Alpha-2,8-Sialyltransferase 4 (ST8SIA4). The haplotype-based analysis revealed that the haplotype (T + TG) with the presence of the nrTE is found with 15 copies in CTRL and 2 in SCZ; indeed, there was a strong association (pval = 6.86 × 10^−5^) between the presence of the nrTE and the absence of the considered trait (schizophrenia). Therefore, we could hypothesize that the haplotype with the nrTE has a protective role against the disease. Further in vitro or in vivo experiments could elucidate this relationship.

The other haplotype (GC-TTI) is characterized by the absence of the AluYb7 on chr4:23511024: indeed, this nrTE is completely absent in CTRL samples and present, only in heterozygous condition, in 7 SCZ patients. Therefore, our hypothesis is that the presence of the element is putatively related with an increased risk of developing schizophrenia. Moreover, the Alu is located in the promoter of MIR548AJ2, one of the 108 genome-wide significant loci for schizophrenia reported by Ripke and colleagues^66^.

As highlighted in the introduction, TEs can act in *cis*, for example, by altering the expression of a gene or by having an impact on its alternative splicing. Therefore, we also looked at the potential role of our 38 significantly different nrTEs by comparing them with the lists produced by Cao and colleagues^46^ based on the GTEx dataset. We found that 27 nrTEs act as eQTLs in different tissues, with 7 showing a putative eQTL effect in the brain. For instance, the AluYb3a1 on chr9:91099740 acts as eQTL in the frontal cortex and is located in the terminator of SPIN1 (Spindlin 1). Moreover, 13 nrTEs act as sQTLs, two of them in the brain: the AluYg6 chr11:76990585 and the AluYb7 on chr2:36476695. These two Alus are located in the third intron of GDPD4 (Glycerophosphodiester Phosphodiesterase Domain-Containing Protein 4) and in the terminator of the CRIM1 gene, respectively. Interestingly, the AluYb7 on chr2:36476695 acts as sQTL in the frontal cortex, and CRIM1 encodes for the cysteine-rich neuron motor 1 protein, which is developmentally regulated and involved in CNS development and organogenesis^75^.

Finally, recent research suggests that TEs could change the local functional architecture of HARs in schizophrenia and bipolar disorder^29^. Therefore, we checked whether our 38 nrTEs are located near (within 1,000bp) or into known HARs (see Materials and Methods), however no match was detected.

In conclusion, our analysis provides the first overview of nrTEs possibly related to an increased risk of developing schizophrenia. We have identified 38 nrTEs of interest and highlighted that several of these elements can have a remarkable impact on the expression, alternative splicing and functionality of nearby genes by cross-checking our results with those available in recently published studies. We also defined two haplotypes in which the presence of the nrTE is either protective against the disease or associated with the schizophrenic condition. Our results, as well as those from other papers dealing with nrTEs, are based on presence/absence of TEs, i.e considering them as biallelic markers; future research based on long-read sequencing will also need to include TEs sequence variability. Moreover, we expect that a similar framework applied to a larger cohort of subjects could confirm and possibly extend our results, and experimental validation of the identified nrTEs will elucidate their effective impact on the cognitive genome.

Having identified both reference^25^ and non-reference TEs associated with an increased risk to develop schizophrenia suggests that a neurodevelopmental genetic mechanism is at play in the etiopathogenesis of this complex disorder. Under this hypothesis, and given that TEs controlling for the functional architecture of the neural genome are mostly evolutionarily recent (either human-only or primate-specific), schizophrenia can emerge as a trade-off between our ongoing cognitive evolution and possible molecular flaws of a not yet completed evolutionary process.

## Abbreviations

1KGP: 1000 Genomes Project
CEU: Europeans
CHB: Han Chinese in Beijing
CNS: central nervous system
CTRL: control individuals
DLPFC: dorsolateral prefrontal cortex
eQTL: expression quantitative trait loci
HAR: human accelerated region
ITU: Indian Telugus
LWK: Luhya in Kenya
nrTE: non-reference transposable element
PCA: principal component analysis
SCZ: schizophrenic individuals
sQTL: alternative splicing quantitative trait loci
TE: transposable element
YRI: Yoruba in Nigeria
